# The prognostic effect of LINC00152 for cancer: a meta-analysis

**DOI:** 10.18632/oncotarget.20130

**Published:** 2017-08-10

**Authors:** Fei-Yu Quan, Jun Jiang, Yi-Fan Zhai, Bing Li, Xin-Hua Wu, Wei Nie

**Affiliations:** ^1^ The 425th Hospital of PLA, Sanya, Hainan, China; ^2^ Department of Respiratory Medicine, Shanghai Changzheng Hospital, Second Military Medical University, Shanghai, China; ^3^ Department of Respiratory Medicine, Eastern Hepatobiliary Surgery Hospital, Second Military Medical University, Shanghai, China; ^4^ Department of Internal Medicine, The 425th Hospital of PLA, Hainan, China

**Keywords:** cancer, LINC00152, biomarker, OS

## Abstract

No meta-analysis has been performed to evaluate the association between LINC00152 and the survival of patients with cancers. We thus carried out this study. The online databases, such as PubMed, EMBASE, and the Cochrane controlled trials register, were searched to identify relevant articles. Dichotomous data were analyzed using the odds ratio (OR) as the summary statistic. The association between LINC00152 and survival of cancer was analyzed by pooling the hazard ratio (HR) with its corresponding 95% confidence interval (CI). Nine studies with 862 patients with cancer were included in this meta-analysis. The expression of LINC00152 was not associated with the age of patients (OR = 0.79, 95% CI = 0.55–1.14) and gender (OR = 1.08, 95% CI = 0.74–1.58). However, we found significant positive associations between LINC00152 and lymph node metastasis (OR = 2.54, 95% CI = 1.54–4.18) and TNM stage (OR = 2.32, 95% CI = 1.36–3.93). Furthermore, the expression of LINC00152 was significantly associated with tumor recurrence (OR = 3.32, 95% CI = 1.98–5.57) and shorter OS (HR = 1.94, 95% CI = 1.25–3.02). In conclusion, the results of this meta-analysis suggest that LINC00152 might be a biomarker for shorter OS and tumor recurrence in cancers.

## INTRODUCTION

Long noncoding RNAs (lncRNAs) are defined as RNA transcripts of more than 200 nucleotides in length [1]. Many studies have suggested that lncRNAs were played an important role in tumorigenesis, proliferation, and metastasis in cancer development [2, 3]. For example, Jiang et al. showed that lnc-epidermal growth factor receptor (EGFR) linked an immunosuppressive state to cancer by promoting Treg cell differentiation [4]. Koirala et al. demonstrates lncRNA AK023948 and DHX9 as important players in the AKT pathway, and that their upregulation may contribute to breast tumour progression [5]. Li et al. indicated that highly up-regulated in liver cancer (HULC) promotes the phosphorylation of Y-box binding protein 1 through the extracellular signal-regulated kinase pathway, in turn regulates the interaction of YB-1 with certain oncogenic mRNAs [6]. However, the exact underlying molecular mechanism and the clinical implications of lncRNAs were still largely unknown.

Long intergenic non-coding RNA 00152 (LINC00152) was suggested to have oncogenic impacts on several cancers [7–15]. It is located on chromosome 2p11.2, which has a transcript length of 828 nucleotides. Recently, Nötzold et al. found that cells depleted of LINC00152 arrested in prometa phase of mitosis and showed reduced Hela cell viability [16]. In RNA affinity purification (RAP) studies, the researchers indicated that LINC00152 interacted with a network of proteins which were associated with M phase of the cell cycle [16]. Chen et al. found that the expression of LINC00152 was significantly associated with tumor invasion depth, lymph node metastasis, and higher tumor-node-metastasis (TNM) stage in gastric cancer [11]. Another group also found that LINC00152 expression was correlated with higher TNM stage, larger tumor size, and lymph node metastasis in lung cancer [7]. In addition, Yu et al. suggested that the increased expression of LINC00152 was significantly correlated with T stage, N stage, TNM stage, and invasion in tongue squamous cell carcinoma [4]. Therefore, we supposed that LINC00152 might influence the survival of patients with cancers. However, no meta-analysis has been performed to evaluate the association between LINC00152 and the survival of patients with cancers. We thus carried out this study.

## RESULTS

### Study characteristics

**Figure 1:**
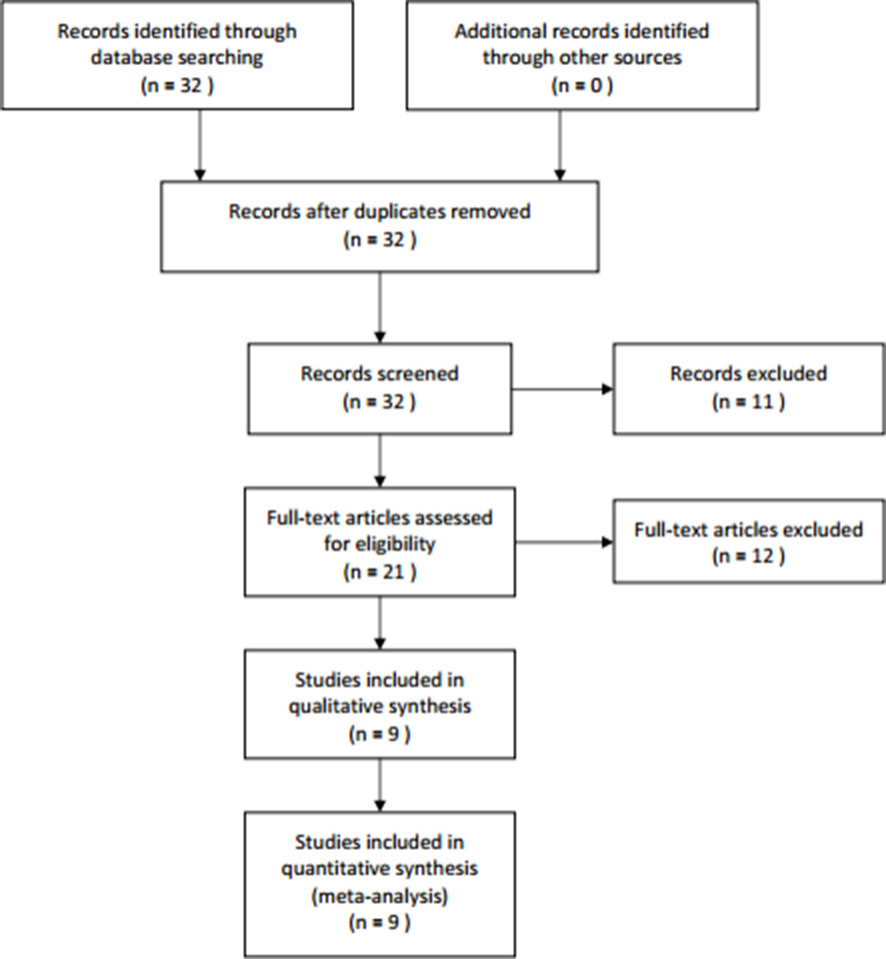
Flow diagram of study identification

Figure 1 showed the process of identifying relevant studies. Thirty-two studies were found in the initial search. After a detailed evaluation, 23 studies were excluded. Finally, 9 studies with 862 patients with cancer were included in this meta-analysis. Colorectal cancer, gastric cancer, renal cell carcinoma, gallbladder cancer, lung cancer, hepatocellular carcinoma, and tongue squamous cell carcinoma were investigated in the original studies. Table 1 showed the characteristics of the included studies. Only 4 studies could provide the data of overall survival (OS).

**Table 1:** Characteristics of the included studies

**First**		**Gender**		**Tumor**	**Samle**			
**author**	**Year**	**(male/female)**	**Site of cancer**	**Stage**	**size**	**Outcome**	**Co-variants**	**NOS**
Qiu	2015	NA	Colorectal cancer	II-III	160	OS	No	7
Chen	2016	69/28	Gastric cancer	I-IV	97	OS	Tumor invasion depth, Lymphatic metastasis, TNM stage	8
Wu	2016	48/29	Renal cell carcinoma	I-III	77	OS	TNM stage, Fuhrman grade	8
Yue	2016	65/69	Colorectal cancer	II-III	134	OS, DFS	TNM stage, AJCC stage	8
Cai	2017	9/26	Gallbladder cancer	I-IV	35	OS*	NA	6
Chen	2017	24/36	Lung cancer	I-III	60	OS*	NA	8
Deng	2017	14/58	Hepatocellular carcinoma	I-IV	72	OS*	NA	7
Wang	2017	27/18	Renal cell carcinoma	I-IV	45	OS*	NA	6
Yu	2017	NA	Tongue squamous cell carcinoma	I-IV	182	OS*	NA	6

DFS, disease-free survival; OS, overall survival; NOS, Newcastle-Ottawa Scale; NA, not available.

*no data of OS could be extracted.

### Results of the meta-analysis

**Figure 2:**
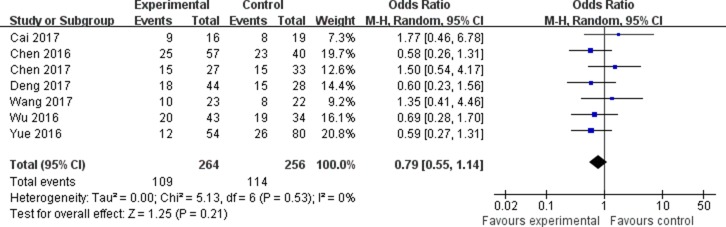
Forest plot (random effects model) describing the association of the LINC00152 with age of patients The expression of LINC00152 was not associated with the age of patients.

**Figure 3:**
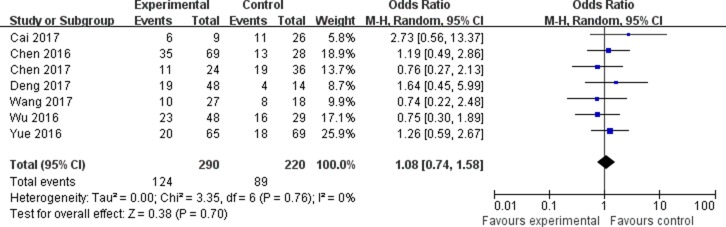
Forest plot (random effects model) describing the association of the LINC00152 with gender of patients The expression of LINC00152 was not associated with the gender of patients.

**Figure 4:**
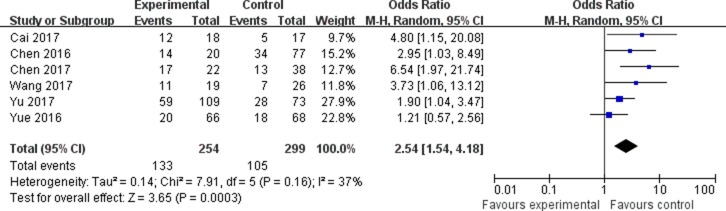
Forest plot (random effects model) describing the association of the LINC00152 with lymph node metastasis The expression of LINC00152 was significantly associated with the lymph node metastasis.

**Figure 5:**
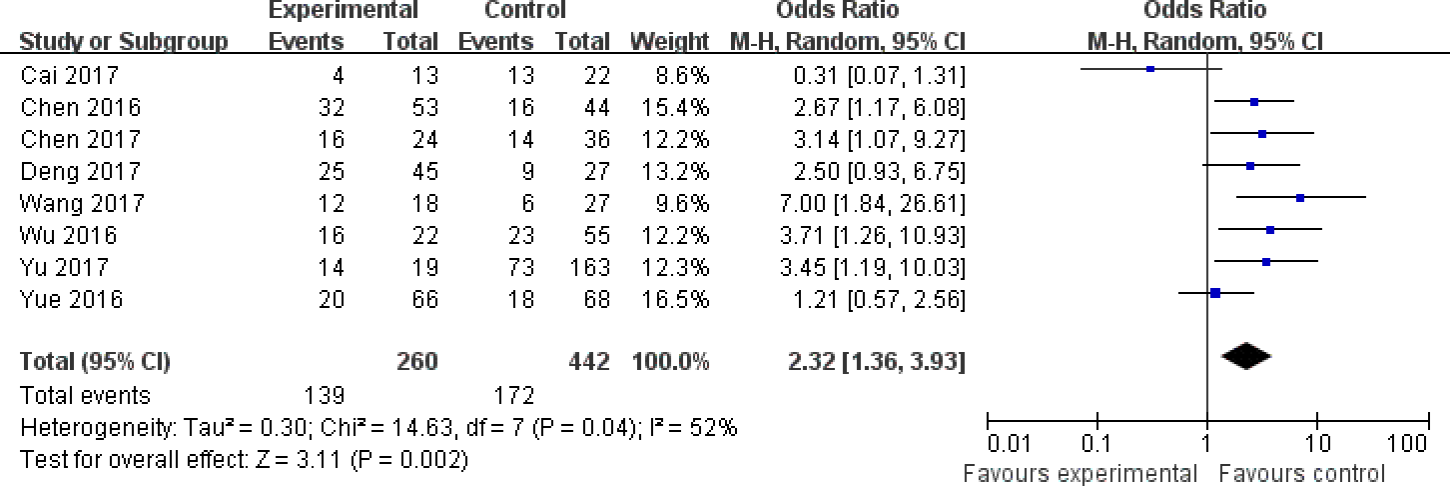
Forest plot (random effects model) describing the association of the LINC00152 with TNM stage The expression of LINC00152 was significantly associated with TNM stage.

**Figure 6:**
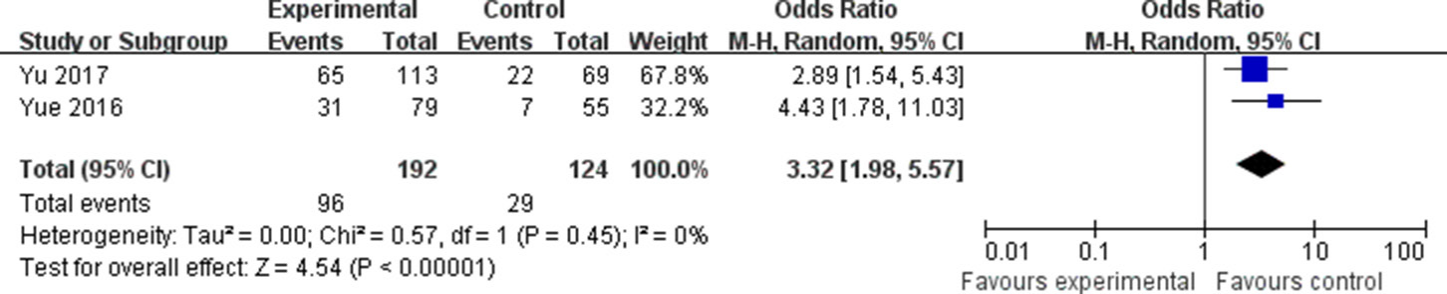
Forest plot (random effects model) describing the association of the LINC00152 with tumor recurrence The expression of LINC00152 was significantly associated with tumor recurrence.

**Figure 7:**
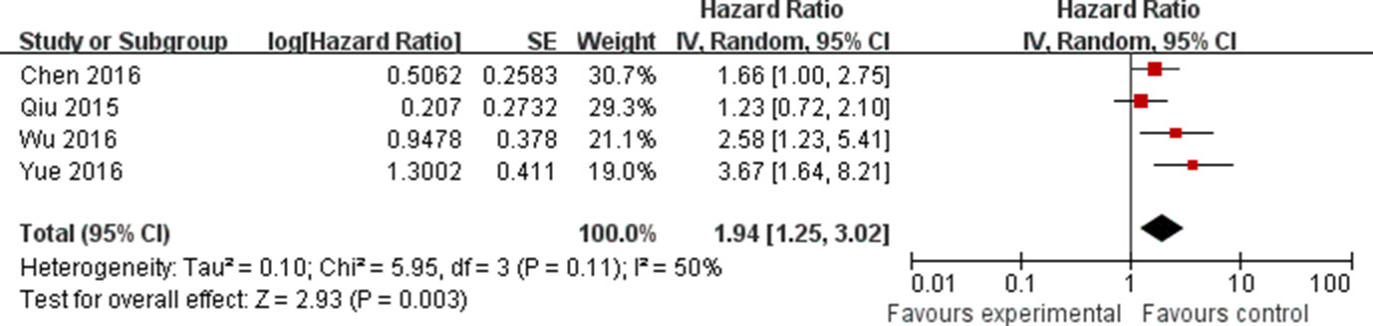
Forest plot (random effects model) describing the association of the LINC00152 with overall survival of cancer The expression of LINC00152 was significantly associated with overall survival of cancer.

The association between the expression of LINC00152 and clinicopathological parameters are shown in Table 2. As shown in Figure 2, the expression of LINC00152 was not associated with the age of patients (OR = 0.79, 95% CI = 0.55–1.14). In addition, no significant association was found between the expression of LINC00152 and gender (OR = 1.08, 95% CI = 0.74–1.58; Figure 3). However, we found significant positive associations between LINC00152 and lymph node metastasis (OR = 2.54, 95% CI = 1.54–4.18; Figure 4) and TNM stage (OR = 2.32, 95% CI = 1.36–3.93; Figure 5). Furthermore, the expression of LINC00152 was significantly associated with tumor recurrence (OR = 3.32, 95% CI = 1.98–5.57; Figure 6) and shorter OS (HR = 1.94, 95% CI = 1.25–3.02; Figure 7).

**Table 2:** The association between LINC00152 and clinicopathological parameters

	Test of association		Heterogeneity	
	OR/HR (95% CI)	*P* Value	**I**^2^ **(%)**	*P* Value
Age	0.79 (0.55–1.14)	0.21	0	0.53
Gender	1.08 (0.74–1.58)	0.70	0	0.76
Lymph node metastasis	2.54 (1.54–4.18)	0.0003	37	0.16
TNM stage	2.32 (1.36–3.93)	0.002	52	0.04
Tumor recurrence	3.32 (1.98–5.57)	< 0.00001	0	0.45
OS	1.94 (1.25–3.02)	0.003	50	0.11

OS, overall survival; HR, hazard ratio; OR, odds ratio; CI, confidence interval.

## DISCUSSION

Many evidence suggested that LINC00152 may participate in the carcinogenesis of cancers [17]. Thus, we conjectured that LINC00152 could change the prognosis of patients with cancers. To our knowledge, this was the first meta-analysis to evaluate the association between the expression of LINC00152 and clinicopathological parameters in cancers. In the present meta-analysis, we found that LINC00152 was significantly associated with lymph node metastasis and TNM stage. Chen et al. found that LINC00152 overexpression could facilitated gastric cancer cell proliferation by accelerating the cell cycle [14]. Cai et al. suggested that LINC00152 could promote cell migration, invasion and epithelial–mesenchymal transition (EMT) progression *in vitro* [11]. Ji et al. found that LINC00152 could promote cell proliferation *in vitro* and tumor growth *in vivo* [18]. However, silencing LINC00152 can suppress the cell proliferation and invasion in hepatocellular carcinoma cells [8]. The mechanism investigation suggested that LINC00152 inhibited the E-cadherin expression via interacting with EZH2 and promoted the Epithelial-mesenchymal transition (EMT) phenomenon in HCC cells [8]. These data might explain why the patients with high level of LINC00152 showed lymph node metastasis and TNM stage. Furthermore, we found significant positive associations between LINC00152 and tumor recurrence and shorter OS of patients with cancer. Thus, LINC00152 might be a potential biomarker in patients with cancer. The doctors should pay more attention to the cancer patients with high expression of LINC00152. However, Qiu did not found LINC00152 was a significant predictor of survival in colorectal cancer [16]. Thus, future studies with colorectal cancer patients are requested to determine this issue.

Some studies investigated the clinical implications of LINC00152 in cancers. Li et al. indicated that plasma levels of HULC and LINC00152 could be used to diagnose hepatocellular carcinoma [19]. Yang and colleagues suggested that serum H19 and LINC00152 might be potential biomarkers for diagnosis of gastric cancer [20]. Yue et al. found that LINC00152 might be a prognostic indicator of oxaliplatin responsiveness in colon cancer patients [21]. In this study, we found that LINC00152 may be a biomarker of the prognosis of cancers. Thus, detecting of LINC00152 might help doctors to manage their patients.

Some limitations of this meta-analysis should be acknowledged. First, only 4 studies could provide the survival data and only 1 study could provide the disease-free survival data. The sample size recruited in the analysis of overall survival was too small, which could not provide statistical power to make significant conclusion. Second, no study from other countries was included in our study. Third, only studies which were indexed by the selected databases were included for data analysis. Fourth, biomarkers are specific for different cancer types or cancer subtypes, especially for LncRNA, because many LncRNAs function in a cell-type-specific way [18]. However, we could not perform a subgroup analysis in a specific cancer or according to the ethnicity due to limited data. We also could not do an *in vitro* experiment. Thus, more studies from other countries are needed to confirm the results from this meta-analysis.

In conclusion, the results of this meta-analysis suggest that LINC00152 might be a biomarker for shorter OS and tumor recurrence in cancers.

## MATERIALS AND METHODS

### Publication search

The online databases, such as PubMed, EMBASE, and the Cochrane controlled trials register, were searched to identify relevant articles published up to MAY 2017 in any language. The electronic search included the terms: LINC00152 and “cancer or carcinoma or neoplasm or tumor”. We also reviewed the reference lists of original reports and reviews. Country was not restricted in this search.

### Inclusion and exclusion criteria

The included studies should meet the following criteria: (1) the study should assessed the association between LINC00152 and the clinicopathological parameters of cancers; (2) cancer should be diagnosed according to histopathological evaluation. The study should be excluded: (1) the study was relevant to cancer or LINC00152; (2) the study was an animal study; (3) the study was a review or abstract.

### Data extraction and quality assessment

Two authors reviewed and extracted the data from original studies independently. The following data were extracted: the first author’s name, year, gender of the patient, site of cancer, tumor stage, sample size, outcome, and co-variants. We used the Newcastle-Ottawa Scale (NOS) to assess the methodological quality of included studies [22].

### Statistical analysis

Dichotomous data were analyzed using the odds ratio (OR) as the summary statistic. The association between LINC00152 and survival of cancer was analyzed by pooling the HR with its corresponding 95% CI. The heterogeneity investigted by using the chi-squared based Q-statistic test. The random-effects model was used to analyze the pooled HRs. If the number of included studies was more than 10, funnel plot was used to analyze the publication bias. All the *P*-values were determined by a 2-sided test. All statistical analyses were conducted using RevMan 5.1 software (Nordic Cochrane Center, Copenhagen, Denmark).
